# Involvement of the Tetraspanin 2 (*TSPAN2*) Gene in Migraine: A Case-Control Study in Han Chinese

**DOI:** 10.3389/fneur.2018.00714

**Published:** 2018-09-11

**Authors:** Jie Fang, Xiaodong Yuan, Xingkai An, Hongli Qu, Chen Wang, Ganji Hong, Liangcheng Zheng, Kehui Yi, Shuai Chen, Xinrui Wang, Qilin Ma

**Affiliations:** ^1^Department of Neurology, The First Affiliated Hospital of Xiamen University, Xiamen, China; ^2^Department of Gynaecology and Obstetrics, Xiamen Maternal and Child Health Care Hospital, Xiamen, China; ^3^Graduate School of Fujian Medical University, Fuzhou, China; ^4^Department of Neurology, Zhongshan Xiamen Hospital, Fudan University, Xiamen, China; ^5^Fujian Medical University, Fuzhou, China; ^6^State Key Laboratory for Medical Genomics, Shanghai Institute of Hematology, Rui-Jin Hospital Affiliated to School of Medicine, Shanghai Jiao Tong University, Shanghai, China

**Keywords:** migraine, Tetraspanin 2, TSPAN2, SNP, genotype-phenotype analysis

## Abstract

Tetraspanin 2 (TSPAN2) belongs to the tetraspanin superfamily. Previous studies have identified significant associations of the *TSPAN2* single nucleotide polymorphisms (SNPs) rs12134493 and rs2078371 with migraine in Western populations; however, these associations need to be confirmed in the Chinese Han population. In addition, we carried out further studies to see whether *TSPAN2* is associated with susceptibility to migraine to provide new clinical evidence. A case-control study (425 patients with migraine and 425 healthy controls) in a Chinese Han population was performed to evaluate the associations between migraine and *TSPAN2* via a genotype-phenotype analysis between *TSPAN2* and clinical symptoms. The SNP rs2078371 was found to be significantly associated with migraines especially in migraines without aura (MO) and in female patients. Meta-analysis revealed that the A allele of rs12134493 was significantly associated with migraines (OR = 1.14, *P* = 0.0001). Our findings suggested that *TSPAN2* is a potential susceptibility factor for migraines. To confirm our results, a large-scale Chinese Han population study should be conducted. Considering that these two SNPs have not been definitively shown to affect *TSPAN2* or to regulate nearby genes in this genomic region, the biological function and molecular mechanism of *TSPAN2* in migraine should be further explored.

## Introduction

Migraine is a complex neurovascular disorder with symptoms including severe headache, nausea, vomiting, photophobia, and phonophobia (1). Almost 14% of the adult population worldwide, and 9.3% of Chinese population in mainland China, are affected by this disease (2, 3). Migraines can be divided into two main subtypes according to The International Classification of Headache Disorders, 3rd edition (ICHD-III) (4): migraine without aura (MO) and migraine with aura (MA). Although a number of epidemiological studies have discovered that migraine has a strong correlation with genetic polymorphisms (5), its etiology and pathogenesis remain poorly understood because of the problematic differential diagnoses.

Approximately 50% of migraine patients have first-degree relatives with the same disorder, and the heritability estimates reported in twin studies ranged from 34 to 57% (6–8). Based on previous studies and the existing theory, the importance of the genetic basis of migraine was fully recognized and systematically proven. In addition, dozens of susceptibility genes related to migraine have been identified by genome-wide association studies (GWASs) conducted in large case-control cohorts (9–11).

Tetraspanin 2 (TSPAN2) is a less well-characterized member of the tetraspanin superfamily, which constitutes 33 cell surface membrane proteins. However, a potential role of TSPAN2 in oligodendrogenesis and in stabilizing the mature sheath (12) has been demonstrated. Anttila et al. conducted a meta-analysis, including 29 studies with 23,285 migraine patients and 95,425 healthy controls (13). Twelve single nucleotide polymorphisms (SNPs) were identified in relation to migraine susceptibility. Among the SNPs, rs12134493 is located upstream of the *TSPAN2* gene. Meanwhile, the SNP rs2078371 maps close to the *TSPAN2* gene and has been proven to have a significant association with migraine (14).

The Chinese Han people are genetically distinct from other races, with a distinctive lifestyle and different genetic polymorphisms; therefore, our replication study aimed to evaluate the association of the SNPs rs12134493 and rs12134493 with migraine in a Chinese Han population from southern Fujian province, China. Moreover, we investigated the genotype-phenotype association to establish the quantitative correlation, and we assessed the impact of the two SNPs on the migraine phenotype.

## Methods

### Patients and samples

The study was approved by the ethics committee of the First Affiliated Hospital of Xiamen University, and it was conducted using clinical samples from patients with migraine who were treated from February 2013 to February 2016. The control group comprised of 425 non-headache, age and gender-matched healthy volunteers who were recruited from the same regional background (southern Fujian province). All of the patients with migraine were diagnosed as suffering either from MA or MO by two headache specialists after neurological examination, direct interview, computed tomography (CT), or magnetic resonance imaging (MRI), according to the diagnostic criteria set by the ICHD-III (4). In addition, the healthy controls had no personal or family history of chronic headache. This study was carried out in accordance with the code of ethics of the World Medical Association (Declaration of Helsinki) for experiments in humans.

### Genetic analysis

Genomic DNA from all subjects was extracted from whole blood samples using 0.5 M ethylene diamine tetraacetic acid (EDTA) as an anticoagulant, using the QiaAmp DNA Mini Kit (Qiagen, Hilden, Germany). The DNA samples were stored at −20°C. Genotyping of rs12134493 and rs2078371 in *TSPAN2* was performed using the Multiplex SNaPshot technique (Applied Biosystems by Life Technologies, Foster City, CA, USA). PCR amplifications were performed in a final volume of 25 μl, containing 50 mM MgCl_2_, 10 mM dNTP, 1 μM primers, and 5 units of Platinum Taq DNA polymerase. The PCR conditions used were 95°C denaturation for 2 min; followed by 33 cycles at 95°C for 20 s, 55°C for 20 s, and 72°C for 40 s; and a final extension step at 72°C for 5 min. The SNaPshot reaction was performed in a final volume of 5 μl (reaction mix 2.5 μl, PCR products 1.5 μl, and probe mix 1.0 μl). DNA sequencing was performed in a final volume of 10 μl containing 1 μl of SNaPshot purified product, 8.5 μl of deionized formamide, and 0.5 μl GeneScan-120 LIZ Size Standard, using an ABI PRISM 3730 DNA Sequencer (Applied Biosystems by Life Technologies). All sequence analyses were performed using GeneMapper4.0 DNA Sequencing Analysis software.

### Statistical analysis

SHEsis software (http://analysis.bio-x.cn/myAnalysis.php) was used to calculate the Hardy-Weinberg equilibrium (HWE), linkage disequilibrium blocks, and haplotype association risk (15). SPSS version 20.0 (IBM Corp., New York, NY, USA) was used for all the statistical analyses. The chi-squared test or *t*-test was used to compare age and gender among the groups; allele and genotype frequencies were compared using the chi-squared test. The genotypes of each SNP were also assessed according to dominant [AA vs. AB + BB (A, major allele; B, minor allele)] or recessive (AA + AB vs. BB) models. The odds ratio (OR) and its corresponding 95% confidence interval (CI) were used as estimates of the association between case-control status and each polymorphism using an unconditional multiple logistic regression model, both with and without adjustment for sex and age. The genotype-phenotype correlation was estimated using the OR and 95% CI from logistic regression analyses with adjustment for sex and age. The R software was used to perform the meta-analysis in the META package (16). The heterogeneity of intervention effects among the studies was evaluated using Cochrane's test. Significant heterogeneity was considered if *P* < 0.05 with the *I*^2^ statistic test, where the *I*^2^ cutoff values were 25, 50, and 75% for low, moderate, and high heterogeneities, respectively. *P* values were adjusted for multiple testing using the Bonferroni correction; therefore, we used *P* < 0.05/5 = 0.01 as a threshold for significance.

## Results

### Subject demographic analysis

The characteristics of the patients with migraine and the controls involved in this study are presented in Table 1. The subjects' demographics did not differ significantly between the patients with migraine and controls, with respect to gender and age (*P* = 0.6528/0.8959). The mean ± SD for age was 36.18 ± 10.39 years for the patients with migraine (range 14–70) and 36.17 ± 8.94 years for the controls (range 14–70). Patients were classified into two groups, including MA (*N* = 71, 52 females and 19 males; mean age: 32.48 ± 11.09) and MO (*N* = 354, 301 females and 53 males; mean age: 36.92 ± 10.11).

**Table 1:** Demographic characteristics of the study subjects.

**Characteristics**	**Control**	**Migraine**	***P*-value**	**MA**	***P*-value**	**MO**	***P*-value**
	**(*n* = 425)**	**(*n* = 425)**	**Migraine vs. control**	**(*n* = 71)**	**MA vs. control**	**(*n* = 354)**	**MO vs. control**
Gender (male/female)	78 (18.4%)/ 347 (81.6%)	72 (16.9%)/ 353 (83.1%)	0.6528	19 (26.8%)/ 52 (72.2%)	0.1358	53 (15.0%)/ 301 (85.0%)	0.246
Age (years) Mean ± SD	36.17 ± 8.94	36.18 ± 10.39	0.8959	32.48 ± 11.09	**0.000676**	36.92 ± 10.11	0.1723
							
Age of onset (years) Mean ± SD		26.98 ± 9.55		24.49 ± 9.30	**0.01548** (MA vs. MO)	27.48 ± 9.53	0.4669 (MO vs. Migraine)
					**0.0401** (MA vs. Migraine)		
Range (Age)	14–70	14–70		14–70		14–66	

MA, migraine with aura; MO, migraine without aura; n, number; SD, standard deviation; vs, versus.

*Significant P-values are in bold*.

### Association of *TSPAN2* SNP (rs12134493) with migraine

The CC, CA, and AA genotype frequencies of the *TSPAN2* SNP rs12134493 were 85.7, 14.1, and 0.2% in the controls and 84.5, 15, and 0.5% in the patients, respectively. The allele frequencies of the *TSPAN2* SNP rs12134493 were 92.7% (C) and 7.3% (A) in the controls and 92% (C) and 8% (A) in the patients (Table 2). The genotype frequencies of the controls and patients were both consistent with the HWE (*P* = 0.366/0.635). The distribution of the *TSPAN2* CA polymorphism was not significantly different between the patients and the controls, before and after adjustment for age and sex (crude OR = 1.082, *P* = 0.687/adjusted OR = 1.128, *P* = 0.543), as it was for the AA polymorphisms (crude OR = 2.028, *P* = 0.564/adjusted OR = 1.882, *P* = 0.612) and the A allele (crude OR = 1.105, *P* = 0.584/adjusted OR = 1.143, *P* = 0.471) (Table 2). In addition, there was no association between migraine subtypes (MA/MO) or gender subtypes and the *TSPAN2* SNP rs12134493 in this population (Tables 3, 4).

**Table 2:** Genotype and allelic frequencies of the *TSPAN2* SNPs in patients and controls.

**SNP**		**Genotypes and alleles**	**Migraine *n*%**	**Control *n*%**	**Crude**	**Crude**	**Adjusted**	**Adjusted**
					**OR (95% CI)**	***P*-value**	**^a^OR (95% CI)**	***P*-value**
*TSPAN2* rs12134493	Genotype	CC CA	359 (84.5%) 64 (15%)	364 (85.7%) 60 (14.1%)	1 1.082 (0.739–1.583)	0.687	1 1.128 (0.766–1.660)	0.543
		AA	2 (0.5%)	1 (0.2%)	2.028 (0.183–22.463)	0.564	1.882 (0.164–21.645)	0.612
	Dominant model	AA	2 (0.5%)	1 (0.2%)	1		1	
		CA+CC	423 (99.5%)	424 (99.8%)	0.499 (0.045–5.522)	0.571	0.540 (0.047–6.205)	0.621
	Recessive model	CC	359 (84.5%)	364 (85.7%)	1		1	
		CA + AA	66 (15.5%)	61 (14.3%)	1.097 (0.752–1.600)	0.631	1.141 (0.778–1.673)	0.498
	Allele	C	782 (92%)	788 (92.7%)	1		1	
		A	68 (8%)	62 (7.3%)	1.105 (0.773–1.581)	0.584	1.143 (0.795–1.641)	0.471
*TSPAN2* rs2078371	Genotype	TT TC	379 (89.2%) 46 (10.8%)	343 (80.7%) 81 (19.1%)	1 0.514 (0.348–0.759)	**0.001**	1 0.529 (0.356–0.784)	**0.002**
		CC	0	1 (0.2%)	n.a.	n.a.	n.a.	n.a.
	Dominant model	CC	0	1 (0.2%)	1		1	
		TC+TT	425 (100%)	424 (99.8%)	n.a.	n.a.	n.a.	n.a.
	Recessive model	TT	379 (89.2%)	343 (80.7%)	1		1	
		TC+CC	46 (10.8%)	82 (19.3%)	0.508 (0.344–0.749)	**0.001**	0.523 (0.353–0.774)	**0.001**
	Allele	T	804 (94.6%)	767 (90.2%)	1		1	
		C	46 (5.4%)	83 (9.8%)	0.529 (0.364–0.768)	**0.001**	0.523 (0.353–0.774)	**0.002**

*CI, confidence interval; OR, odds ratio; n, number; n.a., not applicable; a, Adjusted for age and sex. Significant P-values are in bold*.

**Table 3:** Genotype and allelic frequencies of the *TSPAN2* SNPs in patients with different migraine subtypes.

**SNP**		**Genotypes and alleles**	**Control *n*%**	**Migraine**		**OR (95% CI)** **MA** **vs.** **Control**	***P*-value** **MA** **vs.** **Control**	**OR (95% CI)** **MO** **vs.** **Control**	***P*-value** **MO** **vs.** **Control**
				**Without aura n%**	**With aura n%**				
*TSPAN2* rs12134493	Genotype	CC CA	364 (85.7%) 60 (14.1%)	299 (84.5%) 53 (15%)	60 (84.5%) 11 (15.5%)	1 1.112 (0.553–2.236)	0.765	1 1.075 (0.721–1.604)	0.722
		AA	1 (0.2%)	2 (0.5%)	0	n.a.	n.a.	2.435 (0.220–26.982)	0.468
	Dominant model	AA	1 (0.2%)	2 (0.5%)	0	1		1	
		CA+CC	424 (99.8%)	352 (99.5%)	71 (100%)	n.a.	n.a.	0.415 (0.037–4.597)	0.474
	Recessive model	CC	364 (85.7%)	299 (84.5%)	60 (84.5%)	1		1	
		CA+AA	61 (14.3%)	55 (15.5%)	11 (15.5%)	1.094 (0.545–2.198)	0.801	1.098 (0.739–1.630)	0.644
	Allele	C	788 (92.7%)	651 (91.9%)	131 (92.3%)	1		1	
		A	62 (7.3%)	57 (8.1%)	11 (7.7%)	1.067 (0.548–2.080)	0.848	0.899 (0.618–1.307)	0.576
*TSPAN2*	Genotype	TT	343 (80.7%)	320 (90.4%)	59 (83.1%)	1		1	
rs2078371		TC	81 (19.1%)	34 (9.6%)	12 (16.9%)	0.861 (0.442–1.677)	0.66	0.450 (0.293–0.691)	**0.000259**
		CC	1 (0.2%)	0	0	n.a.	n.a.	n.a.	n.a.
	Dominant model	CC	1 (0.2%)	0	0	1		1	
		TC + TT	424 (99.8%)	354 (100%)	72 (100%)	n.a.	n.a.	n.a.	n.a.
	Recessive model	TT	343 (80.7%)	320 (90.4%)	59 (83.1%)	1		1	
		TC+CC	82 (19.3%)	34 (9.6%)	12 (16.9%)	0.851 (0.437–1.656)	0.634	0.444 (0.290–0.682)	**0.000203**
	Allele	T	767 (90.2%)	674 (95.2%)	130 (91.5%)	1		1	
		C	83 (9.8%)	34 (4.8%)	12 (8.5%)	0.853 (0.453–1.607)	0.623	0.466 (0.309–0.704)	**0.000259**

*CI, confidence interval; OR, odds ratio; n, number; vs, versus; n.a., not applicable; Significant P-values are in bold*.

**Table 4:** Genotype and allelic frequencies of the *TSPAN2* SNPs in patients with different gender subtypes.

**SNP**		**Genotypes and alleles**	**Control *n*%**	**Migraine**		**OR (95% CI)**	***P*-value**	**OR (95% CI)**	***P*-value**
						**Female vs. control**	**Female vs. control**	**Male vs. control**	**Male vs. control**
				**Male *n*%**	**Female *n*%**				
*TSPAN2*	Genotype	CC	364 (85.7%)	57 (79.2%)	302 (85.5%)	1		1	
rs12134493		CA	60 (14.1%)	15 (20.8%)	49 (13.9%)	0.984 (0.655–1.479)	0.939	1.596 (0.849–3.000)	0.146
		AA	1 (0.2%)	0	2 (0.6%)	2.411 (0.218–26.714)	0.473	n.a	n.a
	Dominant model	AA	1 (0.2%)	0	2 (0.6%)	1		1	
		CA + CC	424 (99.8%)	72 (100%)	351 (99.4%)	0.414 (0.037–4.584)	0.472	n.a	n.a
	Recessive model	CC	364 (85.7%)	57 (79.2%)	302 (85.5%)	1		1	
		CA + AA	61 (14.3%)	15 (20.8%)	51 (14.5%)	1.008 (0.674–1.506)	0.97	1.570 (0.836–2.949)	0.16
	Allele	C	788 (92.7%)	129 (89.6%)	653 (92.5%)	1		1	
		A	62 (7.3%)	15 (10.4%)	53 (7.5%)	1.032 (0.705–1.510)	0.873	1.478 (0.816–2.676)	0.197
*TSPAN2*	Genotype	TT	343 (80.7%)	63 (87.5%)	316 (89.5%)	1		1	
rs2078371		TC	81 (19.1%)	9 (12.5%)	37 (10.5%)	0.496 (0.326–0.753)	**0.001**	0.605 (0.289–1.267)	0.183
		CC	1 (0.2%)	0	0	n.a	n.a	n.a	n.a
	Dominant model	CC	1 (0.2%)	0	0	1		1	
		TC + TT	424 (99.8%)	72 (100%)	353 (100%)	n.a	n.a	n.a	n.a
	Recessive model	TT	343 (80.7%)	63 (87.5%)	316 (89.5%)	1		1	
		TC + CC	82 (19.3%)	9 (12.5%)	37 (10.5%)	0.490 (0.323–0.743)	**0.001**	0.598 (0.285–1.251)	0.172
	Allele	T	767 (90.2%)	135 (93.8%)	669 (94.8%)	1		1	
		C	83 (9.8%)	9 (6.2%)	37 (5.2%)	0.511 (0.342–0.763)	**0.001**	0.616 (0.302–1.25)	0.182

*CI, confidence interval; OR, odds ratio; n, number; n.a., not applicable. Significant P-values are in bold*.

### Association of *TSPAN2* SNP (rs2078371) with migraine

The TT, TC, and CC genotype frequencies of the *TSPAN2* SNP rs2078371 were 80.7, 19.1, and 0.2% in the controls and 89.2, 10.8, and 0 in the patients, respectively. The allele frequencies of the *TSPAN2* SNP rs2078371 were 90.2% (T) and 9.8% (C) in the controls and 94.6% (T) and 5.4% (C) in the patients (Table 2). The genotype frequencies of the controls and patients were both consistent with the HWE (*P* = 0.093/0.238). The distribution of the *TSPAN2* TC polymorphism was significantly different between patients and controls, before and after adjustment for age and sex (crude OR = 0.514, *P* = 0.001/adjusted OR = 0.529, *P* = 0.002), as it was for the C allele (crude OR = 0.529, *P* = 0.001/adjusted OR = 0.523, *P* = 0.002). In addition, an association was detected for the TC genotype between the MO subgroup and the controls (crude OR = 0.450, *P* = 0.000259) and for the T allele (crude OR = 0.466, *P* = 0.000259). Moreover, an association was also detected for the TC genotype between the female subtype and the controls (crude OR = 0.496, *P* = 0.001) and for the C allele (crude OR = 0.511, *P* = 0.001). In conclusion, the C allele of the *TSPAN2* SNP rs2078371 could decrease the risk of migraine, and the T allele would increase such risks. The TC genotype could be considered a risk factor for migraine headaches, especially the MO type, and females with the TC genotype are at a higher risk than males.

### Meta-analysis

**Figure 1:**
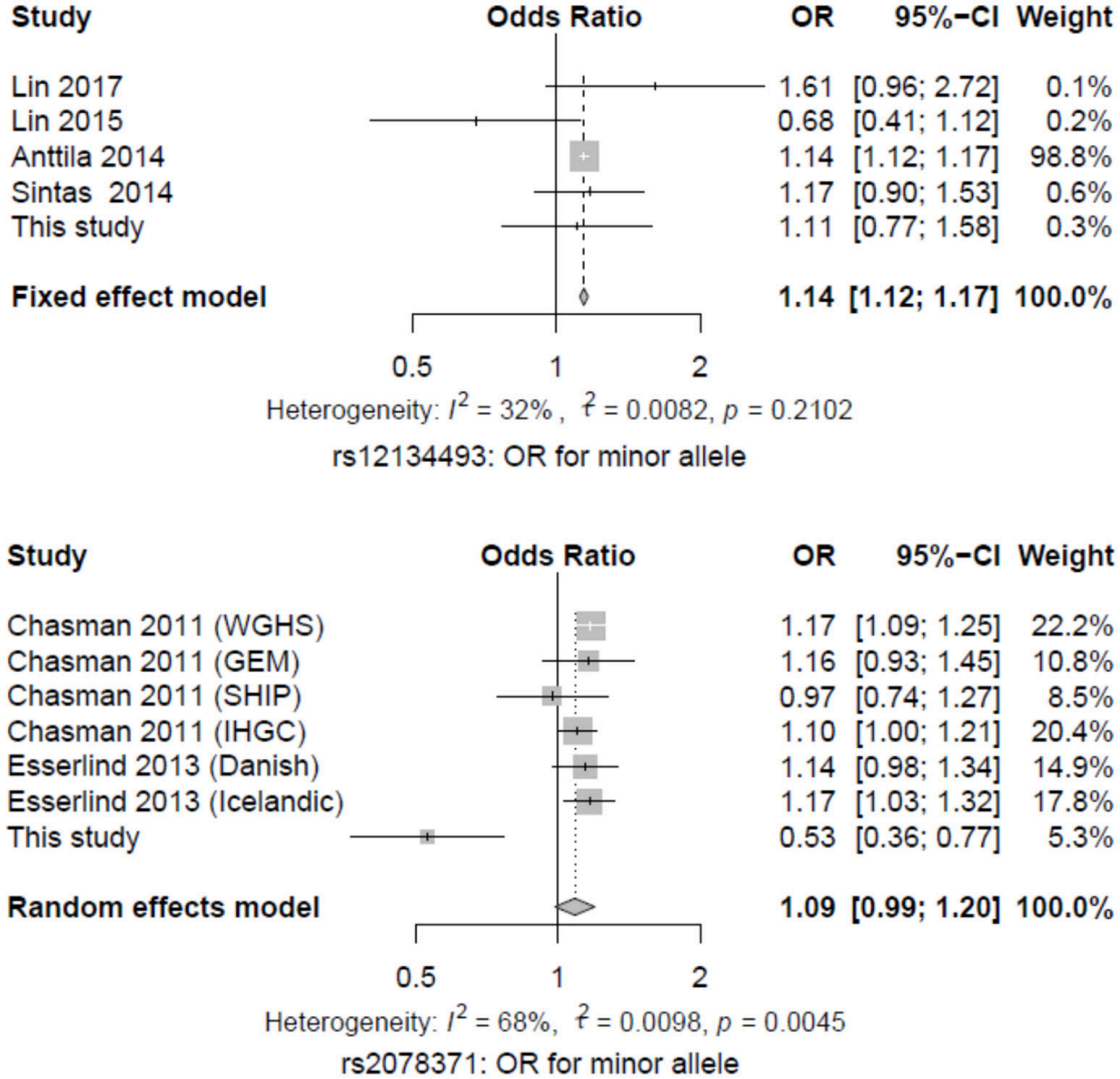
Forest plot of migraine risk associated with the two SNPs in *TSPAN2*. The estimates of ORs and 95% CIs were plotted with a box and a horizontal line.

Eleven studies, including our data and results from the previous reports, were subjected to a meta-analysis using a random-effect or fixed-effect model (8, 13, 14, 17–19). The A allele of the SNP rs12134493 reached genome-wide significance in migraine, with a pooled OR of 1.14 (*P* = 0.0001) and nonsignificant heterogeneity (*I*^2^ = 32%, *P* = 0.2102). While the C allele of the SNP rs2078371 failed to reach the significance level, with a pooled OR of 1.08 (*P* = 0.0954) and a high heterogeneity (*I*^2^ = 68%, *P* = 0.0045) (Figure 1).

### Linkage disequilibrium (LD) tests

The pairwise linkage disequilibrium (LD) between the two SNPs is shown in Table 5. Haplotype analysis in both patients and controls revealed a strong pairwise LD between the rs12134493 and rs2078371 polymorphisms (*D*' = 0.717, *r*^2^ = 0.509). The frequencies of the haplotypes are listed in Table 5, which shows that the frequency of the A-T haplotype is higher in the patients with migraine (*P* = 0.007, OR = 2.728), while the C-C haplotype is more frequent in the healthy controls (*P* = 1.98e-006, OR = 0.099). This result suggested that the A-T haplotype increases susceptibility to migraine, while the C-C haplotype is a protective factor.

**Table 5:** Linkage disequilibrium and distribution of haplotypes of the two SNPs in TSPAN2 between the cases and controls.

	**rs12134493**	**rs2078371**
rs12134493	—	0.717 (D')
rs2078371	0.509 (r^2^)	—
**Haplotype**	**Migraine (%)**	**Control (%)**	χ^2^	**Fisher's** ***p*****-value**	**Pearson's** ***p*****-value**	**OR (95% CI)**
A/C	42.90 (0.050)	52.62 (0.062)	1.049	0.306	0.306	0.805 (0.532–1.220)
A/T	25.10 (0.030)	9.38 (0.011)	7.319	**0.007**	0.007	2.728 (1.280–5.811)
C/C	3.10 (0.004)	30.38 (0.036)	22.672	**1.98E-06**	1.95E-06	0.099 (0.031–0.319)
C/T	778.90 (0.916)	757.62 (0.891)	3.064	0.08	0.08	1.336 (0.965–1.849)

*CI, confidence interval; OR, odds ratio; n.a., not applicable. Significant p-values are in bold*.

### Genotype-phenotype analysis

Genotypes and substantial clinical data from the electronic patient records were available for 425 patients with migraines. None of the migraine features showed a statistically significant correlation with the two SNPs in the migraine group and the MO subgroup (Tables S1, S2). Comparing the minor allele with the maximal allele of the SNP rs12134493 revealed a nominally significant correlation between nausea/vomiting and the MA subgroup (OR = 41.058, *P* = 0.012) (Table 6). However, it did not show a compelling trend after correction for multiple comparisons.

**Table 6:** Genotype-phenotype association of the two SNPs and migraine features in MA group.

**Migraine with aura**	**rs12134493**		**rs2078371**	
**Migraine trait**	**OR (95% CI)**	***P*-value**	**OR (95% CI)**	***P*-value**
Unilateral migraine	0.314 (0.049–2.008)	0.221	0.841 (0.196–3.611)	0.841
Pulsating headache	0.086 (0.003–2.925)	0.173	n.a.	n.a.
Severe headache	1.607 (0.117–22.091)	0.723	3.132 (0.498–19.683)	0.223
Aggravation by physical activity	0.447 (0.054–3.714)	0.456	0.650 (0.105–4.030)	0.644
Nausea/vomiting	41.058 (2.272–749.923)	**0.012**	8.780 (0.543–144.291)	0.128
Phonophobia	0.137 (0.011–1.767)	0.128	0.721 (0.089–5.842)	0.76
Photophobia	3.381 (0.259–44.112)	0.353	1.450 (0.152–13.870)	0.747
Family history	5.051 (0.537–47.519)	0.157	2.645 (0.391–17.888)	0.319
Average age of onset ≤ 27 years	1.043 (0.111–9.825)	0.971	0.411 (0.054–3.134)	0.391
Menstruation-associated^a^	0.112 (0.006–2.010)	0.137	0.274 (0.025–3.003)	0.289

CI, confidence interval; OR, odds ratio. Significant P-values are in bold.

Age and sex-adjusted logistic regression models comparing the minor allele.

a *All female migraine patients*.

## Discussion

The biological function of TSPAN2 in different human diseases and in related tissues remains unclear (20–22). Although studies have revealed that TSPAN2 is involved in tumor metastasis and invasiveness in human lung adenocarcinomas, notably in malignancy (23), further studies are required. In addition, TSPAN2 is highly expressed in oligodendrocyte lineage cells, and it may regulate the differentiation of oligodendrocytes into myelin-forming glia, which suggested that TSPAN2 could have an association with migraine (12, 24). Previous studies, including GWAS findings, identified the SNPs rs12134493 and rs2078371 of *TSPAN2* as being associated with migraine susceptibility. However, these studies focused mainly on Caucasians. The current study evaluated the association of the two SNPs with migraine in a Chinese Han population from southern Fujian province, China.

Anttila et al. proved the association of rs12134493 with migraine in a Western population (13). Subsequently, Sintas et al. replicated this study and identified that rs12134493 was nominally associated with migraine in a Spanish population (16). In contrast to these studies, we failed to replicate the association between the rs12134493 polymorphism and migraine, even in the subtype analysis. In addition, our findings are consistent with those of Lin et al. in another Chinese Han population (19). Furthermore, combining these studies in a meta-analysis showed that the A allele of the SNP rs12134493 reached genome-wide statistical significance for migraine (*P* = 0.0001). The evidence indicated that the association of this variant depends on race and/or ethnicity.

A GWAS study by Chasman et al. and another study by Esserlind et al. identified that the SNP rs2078371 reached genome-wide significance for an association with migraine in Western populations (8). Our findings also supported the view that rs2078371 could be a susceptibility factor for migraine, especially for MO and in females. However, in the present study, this SNP showed a high degree of allelic heterogeneity when compared with those in previous studies. This result is probably caused by the difference in minor allele frequency (MAF) in the Chinese Han population versus the Caucasians, according to 1,000 Genomes Project data (25).

We analyzed the haplotypes of the loci and noticed that the two haplotypes of the four loci of the *TSPAN2* are statistically significant (*P* < 0.01). This suggested that the C-C haplotype is a protective factor for the development of migraine, and it may reduce morbidity risk in this ethnic group. By contrast, the A-T haplotype increases the risk of suffering from migraine by 2.728-fold. In addition, the genotype-phenotype analysis did not provide any evidence of the association of the two susceptibility variants with migraine traits among the migraine group or the MO subtype. Analysis of the MA subtype with the rs12134493 SNP locus revealed a nominal correlation with nausea/vomiting, which did not show a compelling trend after correction for multiple comparisons. Furthermore, the genotype-phenotype analysis might have been underpowered because of the small sample size adopted by this study and the stringent corrections of multiple testing.

In conclusion, we performed a replicate study to identify the associations between the *TSPAN2* SNPs rs12134493 and rs2078371 and migraine in a Chinese Han population. Our findings suggested that rs2078371 could be a potential susceptibility factor for migraine, especially for female sufferers and those with MO. No significant association between rs12134493 and migraine was discovered. The haplotypes analysis showed that the C-C haplotype is a protective factor for migraine, and A-T is a susceptible haplotype. We did not detect any significant influence of these variants on typical migraine symptoms; therefore, their functions remain to be identified. Considering the consistencies and discrepancies between our findings and those of previous studies, a large-scale Chinese Han population study should be conducted to validate the role of *TSPAN2* (26, 27). In addition, considering that these two SNPs have not been shown to affect *TSPAN2* or to regulate nearby genes within this genomic region, we believe that it is necessary to reveal the biological function and molecular mechanism of *TSPAN2* in migraine by integrating genetic, phenotypic, and epigenetic analysis in further research (28).

## Ethics statement

This study conformed to the standards set by the latest revision of the Declaration of Helsinki and was approved by the Ethics Committee of The First Affiliated Hospital of Xiamen University. Written informed consent was obtained from the patients for the publication of this case report.

## Author contributions

JF carried out the molecular genetic studies, performed the statistical analysis, and drafted the manuscript. XA, HQ, XY, CW, GH, LZ, KY and SC carried out the acquisition of data. XW helped to draft the manuscript. QM conceived the study, and participated in its design and coordination. All authors read and approved the final manuscript.

### Conflict of interest statement

The authors declare that the research was conducted in the absence of any commercial or financial relationships that could be construed as a potential conflict of interest.
