# Supplementary material for: Involvement of the Tetraspanin 2 (TSPAN2) Gene in Migraine: A Case-Control Study in Han Chinese
Source: Front Neurol. 2018 Sep 11;9:714. doi: 10.3389/fneur.2018.00714 (PMC6143784; doi:10.3389/fneur.2018.00714)
Supplement: Supplementary file 1 [file Table_1.pdf]

Table S1. Genotype-phenotype association of the two SNPs and migraine features in migraine patients

Migraine	rs12134493		rs2078371	
**Migraine trait**	**OR (95% CI)**	**P-value**	**OR (95% CI)**	**P-value**
Unilateral migraine	1.358 (0.817-2.259)	0.238	1.518 (0.827-2.785)	0.178
Pulsating headache	0.693 (0.332-1.447)	0.329	0.689 (0.283-1.676)	0.412
Severe headache	0.749 (0.351-1.599)	0.456	0.873 (0.371-2.055)	0.757
Aggravation by physical activity	0.908 (0.507-1.627)	0.746	0.970 (0.481-1.959)	0.933
Nausea/vomiting	1.079 (0.524-2.220)	0.837	0.598 (0.207-1.724)	0.341
Phonophobia	0.664 (0.280-1.575)	0.353	1.022 (0.346-3.014)	0.969
Photophobia	1.827 (0.758-4.401)	0.179	1.477 (0.500-4.361)	0.48
Family history	1.575 (0.843-2.941)	0.154	1.540 (0.726-3.269)	0.261
Average age of onset ≤ 27 years	0.791 (0.433-1.445)	0.446	1.036 (0.512-2.098)	0.921
Menstruation-associated[a]	0.624 (0.346-1.125)	0.117	0.862 (0.423-1.756)	0.682

CI: confidence interval, OR: odds ratio.

Age and sex-adjusted logistic regression models comparing the minor allele.

a: All female migraine patients
