# Supplementary material for: Involvement of the Tetraspanin 2 (TSPAN2) Gene in Migraine: A Case-Control Study in Han Chinese
Source: Front Neurol. 2018 Sep 11;9:714. doi: 10.3389/fneur.2018.00714 (PMC6143784; doi:10.3389/fneur.2018.00714)
Supplement: Supplementary file 2 [file Table_2.pdf]

Table S2. Genotype-phenotype association of the two SNPs and migraine features in MO group

Migraine without aura	rs12134493		rs2078371	
**Migraine trait**	**OR (95% CI)**	**P-value**	**OR (95% CI)**	**P-value**
Unilateral migraine	1.580 (0.906-2.756)	0.107	1.931 (0.948-3.934)	0.07
Pulsating headache	0.816 (0.368-1.807)	0.616	1.142 (0.448-2.913)	0.78
Severe headache	0.582 (0.248-1.368)	0.214	0.686 (0.239-1.973)	0.485
Aggravation by physical activity	0.965 (0.517-1.803)	0.911	0.921 (0.414-2.051)	0.841
Nausea/vomiting	0.740 (0.320-1.710)	0.481	0.353 (0.082-1.516)	0.161
Phonophobia	1.070 (0.377-3.038)	0.898	1.714 (0.421-6.983)	0.452
Photophobia	1.372 (0.485-3.879)	0.551	1.050 (0.271-4.069)	0.944
Family history	1.834 (0.903-3.725)	0.094	2.194 (0.860-5.593)	0.1
Average age of onset ≤ 27 years	0.769 (0.404-1.463)	0.423	0.883 (0.398-1.961)	0.76
Menstruation-associated[a]	0.675 (0.360-1.266)	0.221	0.764 (0.344-1.698)	0.509

CI: confidence interval, OR: odds ratio.

Age and sex-adjusted logistic regression models comparing the minor allele.

a: All female migraine patients
